# Prophylactic Antibiotics for Suction Curettage in Incomplete Abortion

**DOI:** 10.1155/S1064744995000044

**Published:** 1995

**Authors:** Kirk D. Ramin, Susan M. Ramin, Patricia G. Hemsell, Brenda J. Nobles, Molly C. Heard, Vivian B. Johnson, David L. Hemsell

**Affiliations:** 1 Department of Obstetrics and Gynecology University of Texas Southwestern Medical Center Parkland Memorial Hospital 5323 Harry Hines Boulevard Dallas TX 75235-9032 USA; 2 Department of Pharmacy University of Texas Southwestern Medical Center Parkland Memorial Hospital Dallas TX USA

## Abstract

*Objective:* The purpose of this study was to investigate the efficacy of 200 mg of prophylactic doxycycline in preventing pelvic infection after curettage for spontaneous (incomplete) abortion.

*Methods:* A randomized, prospective, double-blinded study was carried out involving 300 women with an incomplete abortion who were given either placebo or 200 mg of doxycycline orally 30–60 min prior to curettage. A hematocrit, WBC count, pregnancy test, syphilis serology, *Neisseria gonorrhoeae* culture, and Micro Trak (monoclonal antibody test, Syba, San Jose, CA) for *Chlamydia trachomatis* were performed. The patients were scheduled for follow-up 2 weeks later. Antibiotic administration for any reason as well as the postoperative infection rate in these women was assessed.

*Results:* Eleven women were excluded from analysis, leaving 289 evaluable. *N. gonorrhoeae* was isolated from 6 (2%) women and *C. trachomatis* from 8 (3%) women, and the syphilis serology was serofast in 4 (1%) women. Endometritis complicated the procedure in 4 women who received placebo and in 1 woman who received doxycycline (*P* = 0.22).

*Conclusion:* Prophylactic doxycycline is not effective in preventing pelvic infection after curettage for spontaneous (incomplete) abortion.


Infectious Diseases in Obstetrics and Gynecology 2:213–217 (1995)
© 1995 Wiley-Liss, Inc.

### Prophylactic Antibiotics for Suction Curettage in Incomplete Abortion

**Kirk D. Ramin, Susan M. Ramin, Patricia G. Hemsell, Brenda J. Nobles, Molly C. Heard, Vivian B. Johnson, and David L. Hemsell**

*Departments of Obstetrics and Gynecology (K.D.R., S.M.R., P.G.H., B.J.N., M.C.H., D.L.H.) and Pharmacy (V.B.J.), University of Texas Southwestern Medical Center, Parkland Memorial Hospital, Dallas, TX*

### Abstract

**_Objective:_ The purpose of this study was to investigate the efficacy of 200 mg of prophylactic doxycycline in preventing pelvic infection after curettage for spontaneous (incomplete) abortion.**

**_Methods:_ A randomized, prospective, double-blinded study was carried out involving 300 women with an incomplete abortion who were given either placebo or 200 mg of doxycycline orally 30–60 min prior to curettage. A hematocrit, WBC count, pregnancy test, syphilis serology, _Neisseria gonorrhoeae_ culture, and MicroTrak (monoclonal antibody test, Syba, San Jose, CA) for _Chlamydia trachomatis_ were performed. The patients were scheduled for follow-up 2 weeks later. Antibiotic administration for any reason as well as the postoperative infection rate in these women was assessed.**

**_Results:_ Eleven women were excluded from analysis, leaving 289 evaluable. _N. gonorrhoeae_ was isolated from 6 (2%) women and _C. trachomatis_ from 8 (3%) women, and the syphilis serology was serofast in 4 (1%) women. Endometritis complicated the procedure in 4 women who received placebo and in 1 woman who received doxycycline ($P = 0.22$).**

**_Conclusion:_ Prophylactic doxycycline is not effective in preventing pelvic infection after curettage for spontaneous (incomplete) abortion.** © 1995 Wiley-Liss, Inc.

Key words

Endometritis, doxycycline, spontaneous abortion, prophylaxis

It is well known that the use of antibiotic prophylaxis for women undergoing vaginal hysterectomy or cesarean delivery reduces the rate of infection and febrile morbidity following these procedures.[1–5] Controversy exists on the efficacy of prophylactic antibiotics for elective terminations of pregnancy. Several randomized clinical studies reported that prophylactic antibiotics decreased infectious morbidity following elective curettage abortions by approximately 50%.[6–8] To date, there are no known published studies addressing the prophylactic use of antibiotics in the clinical setting of suction curettage for spontaneous incomplete abortion. At our institution, women undergoing suction curettage for incomplete abortion were commonly given a 7–10 day course of doxycycline without documentation of patient benefit. We sought to evaluate the efficacy of a single dose of prophylactic doxycycline, compared with placebo, in women undergoing suction curettage for spontaneous incomplete abortion and to establish the postoperative infection rate in these women.

Address correspondence/reprint requests to Dr. Susan M. Ramin, Department of Obstetrics and Gynecology, University of Texas Southwestern Medical Center, 5323 Harry Hines Boulevard, Dallas, TX 75235-9032.

Presented in part at the Infectious Disease Society for Obstetrics and Gynecology, Monterey, CA, August 1994.

Received September 12, 1994
Accepted December 29, 1994

Clinical Study

**TABLE 1. Demographics of 289 women given 200 mg of doxycycline or placebo before suction curettage for spontaneous incomplete abortion**

| | Prophylactic regimen | | | |
|---|---|---|---|---|
| Variable | Doxycycline (N = 137) | | Placebo (N = 152) | |
| Age (years) | 25.1 ± 7.3[a] | | 24.7 ± 6.6 | |
| Gravidity | 2.7 ± 1.8 | | 3.0 ± 1.9 | |
| Parity | 1.3 ± 1.5 | | 1.4 ± 1.5 | |
| Previous abortions (spontaneous or induced) | 0.5 ± 0.8 | | 0.7 ± 1.0 | |
| Race | | | | |
| African-American | 29 | (21)[b] | 43 | (28) |
| Caucasian | 24 | (17) | 21 | (14) |
| Mexican-American | 79 | (58) | 83 | (55) |
| Other | 5 | (4) | 5 | (3) |
| Marital status | | | | |
| Married | 64 | (47) | 82 | (54) |
| Single | 68 | (49) | 61 | (40) |
| Divorced | 4 | (3) | 5 | (3) |
| Unknown | 1 | (1) | 4 | (3) |

[a]Mean ± standard deviation.
[b]Number with percent in parentheses.

### SUBJECTS AND METHODS

All healthy women presenting to the emergency room at Parkland Memorial Hospital who were diagnosed with spontaneous incomplete abortion were invited to participate in this prospective, double-blinded clinical trial and to sign Institutional Review Board-approved consent forms. The women were randomized by sealed envelopes to receive either 1) 200 mg of doxycycline or 2) placebo. Either doxycycline or placebo was taken orally 30–60 min prior to suction curettage. The exclusion criteria included a history of allergic reactions to tetracycline or doxycycline, fever (defined as a temperature >38°C), unstable vital signs, a uterine size >14 weeks, a hematocrit <30%, antibiotic use within the previous 2 weeks, or any medical illness such as diabetes mellitus or cardiac or pulmonary disease.

A medical history was taken, and a complete physical examination was performed. A pregnancy test, hemoglobin, hematocrit, WBC count, syphilis serology, *Neisseria gonorrhoeae* culture, and MicroTrak (monoclonal antibody test, Syba, San Jose, CA) for *Chlamydia trachomatis* were performed. A suction curettage was carried out and the patient was scheduled for follow-up 2 weeks later. The clinical diagnosis of endometritis was based on the following criteria: temperature >38.0°C, abdominal pain, tenderness on one or both sides of the abdomen, uterine tenderness, parametrial tenderness elicited upon bimanual examination, and foul-smelling lochia. Antibiotic administration for any reason was monitored.

Data were analyzed using the Student's t-test, Fisher exact test, chi-squared test, or Mann-Whitney U-test, when appropriate. Significance was defined as $P \leq 0.05$.

### RESULTS

Between November 1992 and June 1993, 300 women were enrolled. After randomization, the following women were excluded from analysis: 3 women in whom the curettage was not performed, 6 who had had recent antibiotic use, 1 who did not receive the assigned study drug, and 1 who underwent an emergent hysterectomy for uterine perforation and hemorrhage. Therefore, 11 women were excluded, leaving 289 (96%) evaluable. The demographics of the study population, which are summarized in Table 1, were comparable.

The historical (medical and gynecologic) information obtained from these women was also comparable. The estimated gestational age determined by bimanual examination (9.5 weeks) was 2 weeks less than the estimated gestational age by last menstrual period (11.5 weeks) in both groups. No significant

TABLE 2. Laboratory results in 289 women given 200 mg of doxycycline or placebo before suction curettage for spontaneous incomplete abortion

| Variable | Prophylactic regimen: Doxycycline (N = 137) | | Placebo (N = 152) | |
|---|---|---|---|---|
| Preoperative WBC count ($\times 10^3$/mm$^3$) | 10.2 ± 3.3[a] | | 10.6 ± 3.4 | |
| Hematocrit (%) | | | | |
| Pre-curettage | 36.5 ± 4.3 | | 36.8 ± 3.1 | |
| Post-curettage | 32.5 ± 4.4 | | 32.8 ± 3.3 | |
| Products of conception | | | | |
| Yes | 122 | (89)[b] | 137 | (90) |
| No | 14 | (10) | 11 | (7) |
| Molar change | 0 | (0) | 1 | (1) |
| Specimen lost | 1 | (1) | 3 | (2) |

[a]Mean ± standard deviation.
[b]Number with percent in parentheses.

differences were identified in the pre- and post-curettage vital signs. The laboratory results are summarized in Table 2. There was not a significant difference in the mean preoperative WBC count or hematocrit or in blood types. The 25 women with no chorionic villi on pathologic examination were followed with serial quantitative human chorionic gonadotropin values. Two of these women were subsequently diagnosed with ectopic pregnancies. The remaining 23 women were felt to have had complete abortions.

The results of laboratory testing for preexisting sexually transmitted diseases are summarized in Table 3. The presence of existing sexually transmitted diseases in our study population did not appear to increase the risk of post-procedureal pelvic infection. None of the women with chlamydia, gonorrhea, or a history of syphilis had infection. One of the women with *N. gonorrhoeae* on culture returned with a cystitis. The women enrolled in this study were evaluated in regard to those infected vs. those not infected. Due to the small number of those infected, no statistical difference was seen in regard to the parameters previously listed.

The infectious and surgical complications were examined in the 289 evaluable women. While there was no significant difference ($P = 0.45$) in regard to postoperative endometritis, cervicitis, or urinary tract infections among the groups, more women in the placebo arm were diagnosed with metritis (4 vs. 1) following the procedure. The only women who required admission for treatment of infection received doxycycline prophylaxis. Nine women were admitted for medical or surgical complications. Seven were admitted and observed for acute blood loss anemia, and of these 1 was transfused. One woman sustained a laceration of the uterine artery from the suction curette requiring blood transfusion and hysterectomy. In addition, 1 woman had a cardiac arrhythmia and another had severe hypertension off medication. One hundred twenty-two (89%) of the patients who received doxycycline and 137 (90%) of the control patients returned for follow-up. Fifteen patients in each arm failed to return for follow-up. The medical records on these 30 women were reviewed, and these women were found to have had either gynecology or family plan-

TABLE 3. Presence (culture or serology) of selected infections in study patients

| | *N. gonorrhoeae* | *C. trachomatis* | Syphilis |
|---|---|---|---|
| Doxycycline (N = 137) | | | |
| Positive | 3 | 2 | 2 |
| Negative | 126 | 123 | 135 |
| Unsatisfactory specimen | 3 | 1 | — |
| Not done | 5 | 11 | — |
| Placebo | | | |
| Positive | 3 | 6 | 2[a] |
| Negative | 145 | 139 | 149 |
| Unsatisfactory specimen | 2 | 2 | — |
| Not done | 2 | 5 | 1 |

[a]Serofast syphilis.

ning visits within a year after the abortion. None of these women reported complications surrounding the suction curettage.

### DISCUSSION

Preventing postoperative infection following abortion has long been the goal of obstetrician-gynecologists. Between 1975 and 1977, 41 women died following spontaneous abortion, with 15 (37%) of these deaths being attributable to sepsis.[9] Reduction in the size of the bacterial inoculum that enters the surgical site, alteration of the "culture medium" at the surgical site with a decreased capacity for the growth of pathogenic bacteria, penetration of operative-site tissues reducing susceptibility to bacterial invasion, and enhanced killing of phagocytized bacteria by WBCs are all possible means by which prophylactic antimicrobials function.[5]

Grimes and colleagues[10] reviewed the prophylactic use of antimicrobials in elective pregnancy terminations and concluded that "despite the methodological limitations, these studies...support the hypothesis that systemic prophylactic antibiotics are effective in lowering morbidity from curettage abortions by about one half." Calculating the costs (direct and indirect) of hospitalization due to postoperative infection, they estimated that prophylaxis would save $2 million annually. These savings do not include additional long-term costs associated with reproductive failure subsequent to pelvic inflammatory disease. Park et al.[11] analyzed 26,332 women who underwent suction-curettage abortions and concluded that prophylactic antibiotics reduced the rate of febrile complications to about one-third that of women who received no prophylaxis. In an era of cost containment, savings of this magnitude lead many authors to recommend antepartum prophylaxis for abortion.

In a prospective, double-blinded, randomized trial of 400 mg of metronidazole vs. placebo in women undergoing elective first-trimester abortions, the postabortal infection rates were 20.4% in the control group and 3.9% in the treatment group ($P < 0.025$).[12] A history of pelvic inflammatory disease was associated with an increased frequency of infection. Several authors have suggested a beneficial effect of prophylaxis only when selection criteria are used as opposed to general administration. These criteria include positive screens for *N. gonorrhoeae*, *C. trachomatis*, *Trichomonas vaginalis*, a history of gonorrhea, and multiple sexual partners.[13–15]

In the current trial, we evaluated the effect of 200 mg of doxycycline in reducing pelvic infection following suction curettage for incomplete abortion. The incidence of febrile morbidity following such a procedure performed in the outpatient setting of a large inner-city hospital was unknown prior to the completion of this study. Endometritis occurred in 1 woman in the treatment group compared with 4 women in the control group ($P = 0.22$). Of the 5 women with pelvic infection, only 1 (from the treatment arm) was admitted for treatment of her infection. A power analysis revealed that approximately 700 women would be needed to achieve significance, provided the current trend continued. Prophylactic doxycycline is not effective in preventing pelvic infection after curettage for spontaneous (incomplete) abortion.

### REFERENCES

1. Hemsell DL, Cunningham FG, Kappus S, Nobles B: Cefoxitin for prophylaxis in premenopausal women undergoing vaginal hysterectomy. Obstet Gynecol 56:629–634, 1980.
2. Hemsell DL, Hemsell PG, Nobles BJ: Doxycycline and cefamandole prophylaxis for premenopausal women undergoing vaginal hysterectomy. Surg Gynecol Obstet 161: 462–464, 1985.
3. Gilstrap LC: Prophylactic antibiotics for cesarean section and surgical procedures. J Reprod Med 33:588–590, 1988.
4. Ramin SM, Cox SM, Hemsell DL, Gilstrap LC: Single-dose cefmetazole versus cefotetan prophylaxis for cesarean section and vaginal hysterectomy. Adv Ther 8:141–147, 1991.
5. Hemsell DL: Prophylactic antibiotics in gynecologic and obstetric surgery. Rev Infect Dis 13(SID):S821–S841, 1991.
6. Krohn K: Investigation of the prophylactic effect of tinidazole on the postoperative infection rate of patients undergoing vacuum aspiration. Scand J Infect Dis 26(S): 101–103, 1981.
7. Sonne-Holm S, Heisterberg L, Hebjorn S, Dyring-Anderson K, Andersen JT, Hejl BL: Prophylactic antibiotics in first-trimester abortions: A clinical, controlled trial. Am J Obstet Gynecol 139:693–696, 1981.
8. Westrom L, Svensson L, Wolner-Hanssen P, Mardh PA: A clinical double-blind study on the effect of prophylactically administered single dose tinidazole on the occurrence of endometritis after first trimester legal abortion. Scand J Infect Dis 26(S):104–109, 1981.

9. Grimes DA, Cates W, Selik RM: Fatal septic abortion in the United States, 1975–1977. Obstet Gynecol 57:739–744, 1981.
10. Grimes DA, Schulz KF, Cates W Jr: Prophylactic antibiotics for curettage abortion. Am J Obstet Gynecol 150: 689–694, 1984.
11. Park TK, Flock M, Schulz KF, Grimes DA: Preventing febrile complications of suction curettage abortion. Am J Obstet Gynecol 152:252–255, 1985.
12. Heisterberg L, Petersen K: Metronidazole prophylaxis in elective first trimester abortion. Obstet Gynecol 65:371–374, 1985.
13. Levallois P, Rioux JE: Prophylactic antibiotics for suction curettage abortion: Results of a clinical controlled trial. Am J Obstet Gynecol 158:100–105, 1988.
14. Morton K, Regan L, Spring J, Houang E: A further look at infection at the time of therapeutic abortion. Eur J Obstet Gynaecol Reprod Biol 37:231–236, 1990.
15. Sorensen JL, Thranov I, Hoff G, Dirach J, Damsgaard MT: A double-blind randomized study of the effect of erythromycin in preventing pelvic inflammatory disease after first trimester abortion. Br J Obstet Gynaecol 99: 434–438, 1992.
